# Association between loneliness and dementia risk: A systematic review and meta-analysis of cohort studies

**DOI:** 10.3389/fnhum.2022.899814

**Published:** 2022-12-01

**Authors:** Luyao Qiao, Gege Wang, Zhenyu Tang, Siqi Zhou, Jun Min, Min Yin, Min Li

**Affiliations:** ^1^Department of Neurology, The Second Affiliated Hospital of Nanchang University, Nanchang, China; ^2^Institute of Neuroscience, Nanchang University, Nanchang, Jiangxi, China

**Keywords:** loneliness, meta-analysis, dementia, cohort study, Alzheimer’s disease

## Abstract

Loneliness has been reported to be associated with an increased risk of dementia; however, the extent of this relationship remains controversial. This study aimed to assess the strength of the relationship between loneliness and dementia using a meta-analysis approach. PubMed, EMBASE, and China National Knowledge Internet databases were systematically searched for potentially included studies from inception up to 17 February 2022. A meta-analysis was performed using a random-effects model to assess pooled relative risks (RRs) and 95% confidence intervals (CIs). A literature search identified 16 cohort studies (published in 15 articles), among which 4,625 dementia cases and 62,345 individuals were selected for further meta-analysis. Loneliness was associated with an increased risk of Alzheimer’s disease (AD) (RR: 1.72, 95% CI: 1.32–2.23; *P* < 0.001) and dementia (RR: 1.23, 95% CI: 1.16–1.31; *P* < 0.00001). However, no significant association between loneliness and risk of mild cognitive impairment (MCI) (RR: 1.34, 95% CI: 0.97–1.87; *P* = 0.080) or vascular dementia (VaD) (RR: 1.01, 95% CI: 0.51–1.99; *P* = 0.973) was observed. Results revealed that loneliness might increase the risk of Alzheimer’s disease and dementia. Early interventions that limit loneliness may reduce risk of dementia and Alzheimer’s disease.

## Introduction

Dementia is a group of acquired clinical syndromes characterized by the progressive decline in cognition along with psychiatric and behavioral alterations of differing extents. The expected prevalence of the disease in the year 2050 is 152 million (Livingston et al., 2020). The incidence of Alzheimer’s disease (AD) and other types of dementia [mild cognitive impairment (MCI), vascular dementia (VaD), or all-cause dementia] have declined; MCI is a clinical stage on the continuum of cognitive decline between “normal aging” and dementia. It is characterized by impairment in cognition that is not severe enough to require help with activities of daily living (ADLs)/Instrumental activities of daily living (IADLs). Over time, the need for disability, health, and social care among the elderly population has increased (Satizabal et al., 2016). Currently, there is no cure for dementia. Prior studies have identified numerous risk factors for the disease; however, non-modifiable risk factors account for 50–70% of those previously identified (Diamond and Woo, 2014; Kuring et al., 2020). Since our understanding of modifiable risk factors is poor (Yeo et al., 2007; Hudson et al., 2012), further identification of modifiable risk factors is important.

Loneliness is experienced across the lifespan and across cultures. Most adults present transient symptoms of loneliness throughout the course of their lives (Victor et al., 2020; Schutter et al., 2021). Symptoms of loneliness and social isolation overlap; however, loneliness has been shown to be independently associated with health outcomes (Holt-Lunstad et al., 2015; Leigh-Hunt et al., 2017). Additionally, previous systematic reviews have found evidence that poor social relationships (i.e., socially integrated lifestyle, social engagement, and social activities) were associated with an increased risk of dementia (Kuiper et al., 2015; Penninkilampi et al., 2018). Moreover, symptoms of loneliness are particularly severe in individuals with mental illness, and have been associated with recovery delays and poor social functioning. In addition, loneliness has been associated with diabetes, hypertension, cardiovascular diseases, and dementia (Momtaz et al., 2012; Petitte et al., 2015).

A prior meta-analysis determined that loneliness is associated with an increased risk of dementia (Lara et al., 2019); however, several newly published articles were not included in the study (Luchetti et al., 2020; Rafnsson et al., 2020; Sundström et al., 2020; Sutin et al., 2020; Shibata et al., 2021; Freak-Poli et al., 2022; Salinas et al., 2022). Therefore, the strength of the association between loneliness and dementia remains unclear. To determine the extent to which loneliness is associated with dementia, additional assessment is needed. Here, we aimed to comprehensively analyze all available cohort studies to assess the association between loneliness and dementia among individuals of the general population.

## Methods

### Data sources, search strategy, and selection criteria

The meta-analysis of observational studies in epidemiology guidelines were applied to guide and report this meta-analysis (Stroup et al., 2000). An electronic searches were performed in PubMed, EMBASE, and the China National Knowledge Internet were used to identify eligible studies published from database inception to 17 February 2022. When performing searches, the following keywords were used: “loneliness” and “dementia.” Additional details regarding search strategies used for each database are shown in Supplementary Table 1. The searches were restricted to human studies, with no restrictions placed on the publication language. Citations of relevant publications were also reviewed to determine if they should be included in the meta-analysis. Unpublished data and additional information were obtained by contacting corresponding authors *via* e-mail. The most recent report was used if multiple studies used the same patient cohort.

Two investigators (LQ and GW) independently performed the literature search and study selection steps. Conflicts between investigators were settled *via* a group discussion until a consensus was reached. Studies were included if they met the following eligibility criteria: (1) cohort study design; (2) an exposure group experienced loneliness at baseline; (3) control group that experienced non-loneliness at baseline; (4) reported AD, MCI, dementia, or VaD as outcomes post-follow-up; and (5) patients followed-up >1 year.

### Data collection and quality assessment

Two investigators (LQ and GW) independently collected the following information: first author’s name, publication year, location, sample size, female proportion, follow-up duration, mean age, or age range, loneliness measurement, reported outcome, number of cases, adjusted factors, and reported effect estimate. The same two investigators assessed the quality of included studies using the Newcastle-Ottawa Scale (NOS), which contains eight items and nine stars, as follows: selection (4 items, which were given a total possible number of 4 stars), comparability (1 item, 2 stars), and outcome (3 items, 3 stars) (Stang, 2010). Studies given 8 or 9 stars were considered to be of high quality. Discrepancies regarding data collection and quality assessment were resolved by a third author (ML) and by referring to the original report.

### Statistical analysis

In each study, the relationship between loneliness and dementia risk was assessed *via* effect estimates and 95% confidence intervals (CIs). Given the cohort study design, hazard ratios were considered equivalent to relative risks (RRs). One article that included data from multiple population-based cohorts, we considered the analysis for each cohort as an independent study and extracted data separately (Freak-Poli et al., 2022). Analyses reporting RRs were maximally adjusted for potential confounders if the studies reported multivariate-adjusted outcome data. τ^2^ was applied to explore heterogeneity, and *I*^2^ was used to assess heterogeneity across included studies. Significant heterogeneity was defined as *P* < 0.10, as calculated using Cochran’s *Q* statistical test (Higgins and Thompson, 2002). If significant heterogeneity was not observed, pooled RRs with 95% CIs were calculated using a fixed-effect model, whereas a random-effects model was applied when a significant degree of heterogeneity was observed to take into account underlying variation among included studies (DerSimonian and Laird, 1986). Sensitivity analysis was performed to assess the robustness of pooled conclusions and explore potential sources of heterogeneity. Subgroup analyses were performed based on the following factors: validated loneliness measurement (yes vs. no), depression adjustment (yes vs. no), length of follow-up (≥10 vs. <10 years), geographical area (United States vs. Asian vs. European), and study quality (high vs. low). Publication bias was assessed *via* a visual inspection of funnel plots and Egger and Begg tests (Begg and Mazumdar, 1994; Egger et al., 1997; Higgins et al., 2003). The “trim and fill” method was applied to adjust for potentially significant publication bias (Duval and Tweedie, 2000). Moreover, population attributable risk (PAR) was calculated when a significant association between loneliness and dementia was observed using the following formula: PAR% = (*P*_*e*_) (RR − 1)/[(*P*_*e*_)(RR − 1) + 1] × 100, where the proportion of individuals exposed to loneliness was defined as *P*_*e*_ RR was obtained from the estimated RRs (Benichou, 2001). All reported *P*-values were two-sided, and those <0.05 were considered significant. Software Review Manager (version 5.3; Cochrane Collaboration, Oxford, UK) and STATA 12.0 (StataCorp, College Station, TX, USA) were used to perform all statistical analyses.

## Results

### Literature search

**FIGURE 1:**
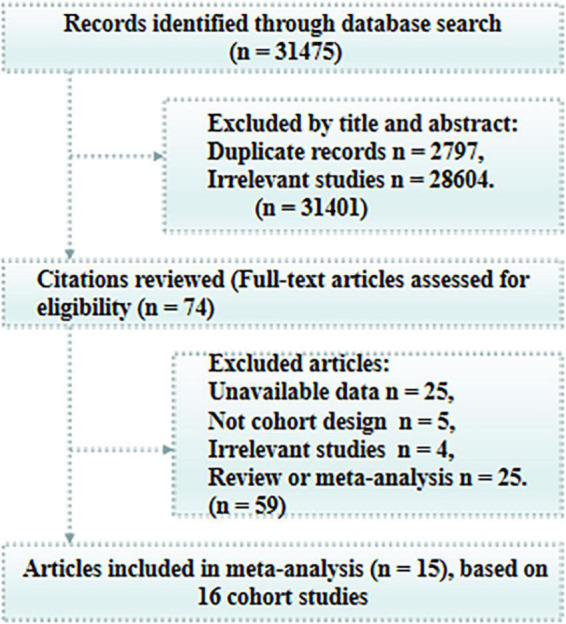
Process of literature search and study selection.

An initial electronic search produced 31,475 potentially relevant articles. Among these, 31,401 articles were excluded due to duplicate records or irrelevance based on a review of titles and abstracts. The remaining 74 potentially eligible articles were retrieved for full-text evaluation, which resulted in the exclusion of 59 due to insufficient data (*n* = 25), a review or meta-analysis design (*n* = 25), a non-cohort design (*n* = 5), or irrelevance (*n* = 4). Because one article reported the Rotterdam study and the Swedish National study separately (Freak-Poli et al., 2022), we considered the analysis for each cohort as an independent study. Finally, the remaining 16 cohort studies (published in 15 articles) were included in the final analysis (Figure 1; Zhang et al., 1999; Tilvis et al., 2004; Wilson et al., 2007; Lobo et al., 2008; Chen et al., 2011; Holwerda et al., 2014; Rawtaer et al., 2017; Zhou et al., 2018; Luchetti et al., 2020; Rafnsson et al., 2020; Sundström et al., 2020; Sutin et al., 2020; Shibata et al., 2021; Freak-Poli et al., 2022; Salinas et al., 2022).

### Study characteristics

Characteristics of articles considered and baseline characteristics individuals included in the analysis are summarized in Supplementary Table 2. Among the 62,345 individuals identified from the 15 articles considered, 4,625 cases were reported. Among the articles considered, three were primarily conducted in the United States (Wilson et al., 2007; Sutin et al., 2020; Salinas et al., 2022), five in Asian countries (China, Japan, and Singapore) (Zhang et al., 1999; Chen et al., 2011; Rawtaer et al., 2017; Zhou et al., 2018; Shibata et al., 2021), and seven in European countries (Tilvis et al., 2004; Lobo et al., 2008; Holwerda et al., 2014; Luchetti et al., 2020; Rafnsson et al., 2020; Sundström et al., 2020; Freak-Poli et al., 2022). The sample size assessed in each article ranged from 650 to 14,411 individuals and the duration of follow-up ranged from 3 to 14 years. Among included articles, the proportion of individuals with dementia ranged from 3.3 to 14.3%, while that of loneliness-associated dementia was approximately 1.5%. Seven articles were considered to be of high quality (Supplementary Table 3; Wilson et al., 2007; Rawtaer et al., 2017; Luchetti et al., 2020; Sundström et al., 2020; Shibata et al., 2021; Freak-Poli et al., 2022; Salinas et al., 2022).

### Loneliness and Alzheimer’s disease risk

**FIGURE 2:**
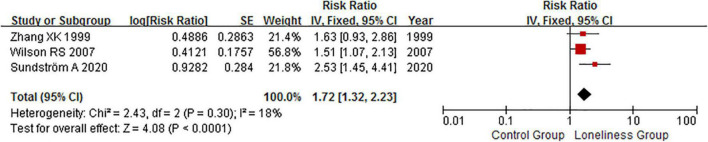
Fixed effects analysis of fully adjusted studies for the association between loneliness and Alzheimer’s disease (AD) risk. The square box in the graph portrays the weight that each study contributed to the analysis. CI, confidence interval; IV, inverse variance; SE, standard error; AD, Alzheimer’s disease.

The association between loneliness and AD risk was reported in three articles, which included 411 AD cases among a total of 3,900 individuals (Zhang et al., 1999; Wilson et al., 2007; Sundström et al., 2020). Among the group, loneliness was associated with an increased risk of AD (RR: 1.72, 95% CI: 1.32–2.23; *P* < 0.001; Figure 2). Further, a non-significant degree of heterogeneity was observed across articles (*P* = 0.30, *I*^2^ = 18%). No significant publication bias for AD was detected using either Begg (*P* = 1.000) or Egger (*P* = 0.503) tests. The PAR of dementia for loneliness in patients with AD was 7%.

### Loneliness and mild cognitive impairment risk

**FIGURE 3:**
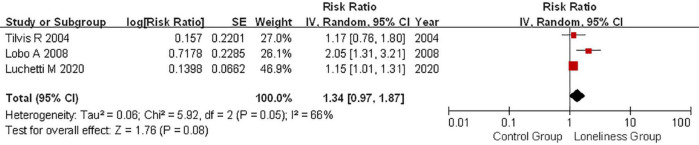
Random effects analysis of fully adjusted studies for the association between loneliness and mild cognitive impairment (MCI) risk. The square box in the graph portrays the weight that each study contributed to the analysis. CI, confidence interval; IV, inverse variance; SE, standard error; MCI, mild cognitive impairment.

An association between loneliness and MCI risk was reported in three articles, among which 821 MCI cases were considered among 16,715 individuals (Tilvis et al., 2004; Lobo et al., 2008; Luchetti et al., 2020). No significant association between loneliness and MCI risk was observed (RR: 1.34, 95% CI: 0.97–1.87; *P* = 0.080; Figure 3). Further, a significant degree of heterogeneity was detected (*P* = 0.05, *I*^2^ = 66%). Publication bias for MCI was not determined to be significant (*P*_Begg_ = 0.296, *P*_Egger_ = 0.499).

### Loneliness and dementia risk

**FIGURE 4:**
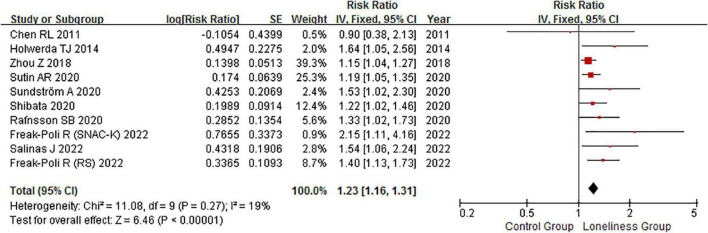
Fixed effects analysis of fully adjusted studies for the association between loneliness and dementia risk. The square box in the graph portrays the weight that each study contributed to the analysis. CI, confidence interval; IV, inverse variance; SE, standard error.

The association between loneliness and dementia risk was reported in nine articles, with 3,648 dementia cases reported among 42,034 individuals (Chen et al., 2011; Holwerda et al., 2014; Zhou et al., 2018; Rafnsson et al., 2020; Sundström et al., 2020; Sutin et al., 2020; Shibata et al., 2021; Freak-Poli et al., 2022; Salinas et al., 2022). Notably, loneliness was associated with an increased risk of dementia (RR: 1.23, 95% CI: 1.16–1.31; *P* < 0.00001; Figure 4). No evidence of heterogeneity across articles (*P* = 0.27, *I*^2^ = 19%) was observed (Supplementary Figure 1). Both the Begg and the Egger test suggested borderline evidence of publication bias (*P*_Begg_ = 0.074, *P*_Egger_ = 0.015). Fixed-effects RR corrected for publication bias using the trim and fill method was (RR: 1.21, 95% CI: 1.13–1.29; *P* < 0.00001) for all articles combined. Correction for potential publication bias therefore did not materially alter the combined risk estimate. The PAR of dementia among those experiencing loneliness was 2%.

### Loneliness and the risk of vascular dementia

One article reported an association between loneliness and VaD, with 157 VaD cases reported among 1,905 included individuals (Sundström et al., 2020). We found no significant association between loneliness and VaD risk (RR: 1.01, 95% CI: 0.51–1.99; *P* = 0.973).

### Subgroup and sensitivity analyses

Subgroup analysis of the relationship between loneliness and dementia risk is shown in Table 1. In all subgroups, loneliness was associated with dementia, except within articles that failed to adjust for depressive symptoms (RR: 1.39, 95% CI: 0.99–1.96; *P* = 0.060; *I*^2^ = 61%). Moreover, the strength of the association between loneliness and dementia was greater among articles with the following characteristics: used validated questionnaires, performed in Europe, and had a follow-up duration ≥10.0 years. The strength of the association between loneliness and dementia risk among subgroups was similar when assessed using quality score. Sensitivity analysis indicated that the results of the present meta-analysis were stable after sequentially removing individual articles (data not shown). Pooled RRs for dementia ranged from 1.22 (95% CI: 1.15–1.29, with Freak-Poli et al., excluded) to 1.27 (95% CI: 1.19–1.35; with Zhou et al., excluded).

**TABLE 1:** Stratified analyses of loneliness and dementia risk.

Group	No. of cohort studies	RR (95% CI)	Heterogeneity test			*P*-value of pooled effect
				
			χ^2^	*P*-value	*I*^2^%	
Loneliness measurement (validated questionnaires)						
Yes	8	1.30 (1.20–1.41)	10.6	0.16	34	<0.00001
No	8	1.17 (1.09–1.26)	6.14	0.52	0	<0.0001
Geographical area						
United States	3	1.25 (1.12–1.40)	2.95	0.23	32	0.0001
Asian	5	1.17 (1.07–1.27)	2.40	0.66	0	0.0004
European	8	1.30 (1.18–1.42)	12.00	0.10	42	<0.00001
Depression adjustment						
Yes	12	1.25 (1.17–1.34)	11.78	0.38	7	<0.00001
No	4	1.39 (0.99–1.96)	7.72	0.05	61	0.06
Mean follow-up (years)						
<10	8	1.22 (1.13–1.32)	11.18	0.13	37	<0.00001
≥10	8	1.24 (1.15–1.34)	8.91	0.26	21	<0.00001
Quality score						
High score > 7	8	1.26 (1.16–1.37)	9.42	0.22	26	<0.00001
Low score ≤ 7	8	1.21 (1.13–1.30)	10.20	0.18	31	<0.00001

CI, confidence interval; RR, relative risk.

## Discussion

We report that loneliness is associated with an increased risk of AD and dementia, while no significant association between loneliness and MCI risk or VaD was observed. Loneliness has previously been identified as a risk factor for premature mortality (Holt-Lunstad et al., 2015; Schutter et al., 2021), adverse biological parameters (e.g., hypertension), health-risk behaviors (including smoking, physical inactivity, and excess alcohol consumption), physical and mental morbidity, and increased health service use (Hawkley et al., 2009; Shankar et al., 2011; Dyal and Valente, 2015). However, the strength of this relationship between loneliness and dementia remains unclear. The current updated meta-analysis considered 4,625 cases of dementia among 62,345 individuals included in 16 cohort studies. Characteristics of both studies and individuals considered ranged widely.

Numerous studies have reported a potential link between loneliness and dementia risk (Wilson et al., 2007; Lobo et al., 2008; Holwerda et al., 2014; Zhou et al., 2018; Luchetti et al., 2020; Rafnsson et al., 2020; Sundström et al., 2020; Sutin et al., 2020; Shibata et al., 2021; Freak-Poli et al., 2022; Salinas et al., 2022); however, several failed to identify a significant association between loneliness and dementia risk (Zhang et al., 1999; Tilvis et al., 2004; Chen et al., 2011; Rawtaer et al., 2017). An earlier meta-analysis found that loneliness was associated with an increased risk of dementia (Lara et al., 2019), a result that was consistent with a study conducted by Luchetti et al. (2020) and the Singapore Longitudinal Aging Study (Rawtaer et al., 2017) included in a prior meta-analysis were limited to those published by November 2018. Since then, seven additional articles on the topic have been published (Luchetti et al., 2020; Rafnsson et al., 2020; Sundström et al., 2020; Sutin et al., 2020; Shibata et al., 2021; Freak-Poli et al., 2022; Salinas et al., 2022). To clarify the putative association between loneliness and dementia, an additional meta-analysis of relevant articles should be conducted.

Studies have demonstrated that loneliness is significantly related to unhealthy behaviors, which may affect cognition or increase risk of cardiometabolic diseases (Lara et al., 2019). Several plausible mechanisms have been put forward including: cognitive activity and neural reserve decreases when neural responses of loneliness are triggered and brain-derived neurotrophic factor is downregulated (Wilson et al., 2007). Further, studies have shown that genetic mechanisms may link loneliness and dementia (Salinas et al., 2017; Hsiao et al., 2018). Loneliness may increase amyloid burden in the elderly, with those individuals carrying APOE4 displaying altered amyloid-related mechanisms (Donovan et al., 2016; Ge et al., 2018). Loneliness is significantly related to depression risk, indicating that depression may affect cognitive decline and dementia (Cacioppo et al., 2010). Our study revealed that the relationship between loneliness and dementia risk may be affected by adjustment for depressive symptoms. This may be explained by the observation that depressive symptoms and social involvement may affect loneliness, which is significantly associated with dementia risk.

The strengths of this study are as follows: (1) it exclusively included cohort studies, which minimized selection and recall biases; (2) the analysis was based on a large sample size, making study findings more robust than those of any individual study; (3) PAR was calculated so that RR and PAR could be used to assess distributions of risk factors; and (4) all included studies were published after 1999 and included relatively complete data.

Several limitations of this study should be acknowledged. First, loneliness was assessed using various questionnaires; therefore, information bias may have affected findings. Second, loneliness was assessed at baseline, which may have affected the assessment of loneliness severity. Third, loneliness was assessed using questionnaires distributed *via* postal mail, and the dementia classification of individuals was not available in medical records. Fourth, factors for which data was adjusted varied among studies included in the meta-analysis. This may have affected the assessment of the progression of dementia. Finally, the analysis was based on published articles and pooled data, a study design that is susceptible to publication bias and restricts the analysis of patient details.

## Conclusion

This study revealed that loneliness may increase risk of developing both AD and dementia. Early interventions that limit loneliness may reduce risk of AD and dementia.

## Data availability statement

The original contributions presented in this study are included in the article/Supplementary material, further inquiries can be directed to the corresponding author.

## Author contributions

ML was the guarantor, conceived, and designed the study. LQ and GW searched the databases and checked these according to the eligible criteria, exclusion criteria, extracted the quantitative data, and wrote the draft of the manuscript. ZT helped develop search strategies. SZ, MY, and JM analyzed the data. All authors contributed in writing, reviewing, or revising the manuscript.
