# Supplementary material for: Association between loneliness and dementia risk: A systematic review and meta-analysis of cohort studies
Source: Front Hum Neurosci. 2022 Dec 1;16:899814. doi: 10.3389/fnhum.2022.899814 (PMC9751343; doi:10.3389/fnhum.2022.899814)
Supplement: Supplementary file 1 [file Data_Sheet_1.DOC]

**MOOSE Checklist**

**Association** **between loneliness and dementia risk: A systematic review and meta-analysis of cohort studies**

Luyao Qiao^a,#^, Gege Wang^a,#^, Zhenyu Tang^a,b^, Siqi Zhou^a^, Jun Min^a,b^, Min Yin^a,b^ and Min Li^a,b^,*

^a^ Department of Neurology, the Second Affiliated Hospital of Nanchang University, Nanchang 330006, PR China

^b^ Institute of Neuroscience, Nanchang University, Nanchang 330006, PR China

* Correspondence to Min Li; Nanchang University; No. 1, Minde Road, Nanchang, Jiangxi 330006, PR China.

Tel: +86 791 86300624;

Fax: +86 791 86292217

1. mail: [minli2012@sina.com](mailto:minli2012@sina.com)

| **Criteria** | | **Brief description of how the criteria were handled in the meta-analysis** |
| --- | --- | --- |
| **Reporting of background should include** | |  |
| √ | Problem definition | Dementia has been recognized as a global health problem and projections suggest the number of people with dementia worldwide may exceed 152 million by 2050. There is an increasing emphasis on the identification of biological and lifestyle influence risk factors for conditions known to predict the ultimate emergence of dementia in order to prevent or delay the onset of dementia. Loneliness has been also associated with diabetes, hypertension, cardiovascular disease, and dementia. However, the role of loneliness in dementia is still controversial. If loneliness may become a modifiable risk factor management in the primary prevention of dementia. To obtain a more comprehensive estimate of the putative influence of the loneliness on dementia, we conducted a meta-analysis of cohort studies to determine the association between loneliness and risk of dementia. |
| √ | Hypothesis statement | loneliness increases risk of dementia. |
| √ | Description of study outcomes | Dementia, AD, MCI, et al. |
| √ | Type of exposure or intervention used | Loneliness |
| √ | Type of study designs used | We included (1) original studies (eg, not review articles, meeting abstracts, editorials, or commentaries); (2) cohort design (eg, not cross sectional design, case-control design). |
| √ | Study population | We restricted the search to human studies. |
| **Reporting of search strategy should include** | |  |
| √ | Qualifications of searchers | The credentials of the two investigators (L.Q. and G.W.) are indicated in the author list. |
| √ | Search strategy, including time period included in the synthesis and keywords | PubMed from 1965 - February 17, 2022  EMBASE from 1974 - February 17, 2022  CNKI from 1990 - February 17, 2022  Keywords: (“loneliness,” “social support,” “social isolation,” “social participation,” “social engagement,” “social disengagement,” “social integration,” “social interaction,” “social withdrawal,” “social capital,” “social contact,” “social influence,” “social vulnerability” and “cognitive function,” “cognitive impairment,” “cognitive decline,” “cognitive deficit*,” “cognition loss*,” “cognitive loss*,” “cognitive abilit*,” “dement*,” “alzheimer*,” “cognition,” “cognitive status,” “cognitive change,” “cognition change,” “cognitive performance,” “cognitive disfunction*”; and “longitudinal studies,” “cohort studies,” and “follow-up studies”). |
| √ | Databases and registries searched | PubMed, EMBASE, and the CNKI |
| √ | Search software used, name and version, including special features | We did not employ a search software. EndNote was used to merge retrieved citations and eliminate duplications |
| √ | Use of hand searching | We hand-searched bibliographies of retrieved papers for additional references, |
| √ | List of citations located and those excluded, including justifications | Details of the literature search process are outlined in the process of literature search and study selection. The citation list is available upon request |
| √ | Method of addressing articles published in languages other than English | We placed no restrictions on language; local scientists fluent in the original language of the article were contacted for translation |
| √ | Method of handling abstracts and unpublished studies | We had contacted a few authors for unpublished studies on the association. |
| √ | Description of any contact with authors | We contacted authors who had conducted multivariate analysis with cognition as a covariate, but the exposure of interest was not loneliness. |
| **Reporting of methods should include** | |  |
| √ | Description of relevance or appropriateness of studies assembled for assessing the hypothesis to be tested | Detailed inclusion and exclusion criteria were described in the methods section. |
| √ | Rationale for the selection and coding of data | Data extracted from each of the studies were relevant to the population characteristics, study design, exposure, outcome, and possible effect modifiers of the association. |
| √ | Assessment of confounding | Restricted the analysis to age- or sex-adjusted estimates only. Conducted sensitivity analyses by eliminating studies that had not adjusted for possible confounders. |
| √ | Assessment of study quality, including blinding of quality assessors; stratification or regression on possible predictors of study results | The Newcastle-Ottawa Scale (NOS) was used to assess the quality of studies. The quality of cohort studies were evaluated in the following three major components: selection of the study group (0-4 stars), quality of the adjustment for confounding (0-2 stars) and assessment of outcome in the cohorts (0-3 stars). A higher score represents better methodological quality. The full score was 9 stars. Studies were graded as the high-quality if they met >7 awarded stars. |
| √ | Assessment of heterogeneity | Heterogeneity of the studies were explored within two types of study designs using Cochrane’s Q test of heterogeneity and *I^2^* statistic that provides the relative amount of variance of the summary effect due to the between-study heterogeneity. |
| √ | Description of statistical methods in sufficient detail to be replicated | Description of methods of meta-analyses, sensitivity analyses, subgroup analyses, and assessment of publication bias are detailed in the methods. |
| √ | Provision of appropriate tables and graphics | We included 1 flow chart, several summary tables and figures. |
| **Reporting of results should include** | |  |
| √ | Graph summarizing individual study estimates and overall estimate | Figure 1, 2, 3, and 4 |
| √ | Table giving descriptive information for each study included | Supplemental tables 1, 2, and 3 |
| √ | Results of sensitivity testing | Table 1 |
| √ | Indication of statistical uncertainty of findings | 95% confidence intervals were presented with all summary estimates, *I^2^* values and results of sensitivity analyses |
| **Reporting of discussion should include** | |  |
| √ | Quantitative assessment of bias | Subgroup analyses indicate heterogeneity in strengths of the association due to most common biases in cohort studies. |
| √ | Justification for exclusion | We excluded studies that had not adjusted for or were standardized by age or sex, a potential confounder, and used different exposure or outcome assessment for the comparison groups. |
| √ | Assessment of quality of included studies | We discussed the results of the subgroup analyses, and potential reasons for the observed heterogeneity. |
| **Reporting of conclusions should include** | |  |
| √ | Consideration of alternative explanations for observed results | We discussed that potential unmeasured confounders such as other chronic diseases may have caused residual confounding, but the measured factors that are correlated with such confounders would have mitigated the bias.  We noted that the variations in the strengths of association may be due to true population differences, or to differences in quality of studies. |
| √ | Generalization of the conclusions | Our meta-analysis suggests that after adjustment for established risk factors, participants with loneliness at baseline were considerably more associated with a greater incidence of dementia risk (combined RR: 1.23, 95% CI 1.16 to 1.31), compared to participants without loneliness. |
| √ | Guidelines for future research | Future studies on the effect of loneliness treatment and modifiable risk factor reduction on dementia risk in loneliness patients are warranted. |
| √ | Disclosure of funding source | This research was supported by the Jiangxi Applied Development Special Fund for Health Science and Technology Development (No. 20181BBG78036). The funders had no roles in the design of the study and collection, analysis, and interpretation of data or manuscript writing. |
