# Supplementary figures and images for: Association between loneliness and dementia risk: A systematic review and meta-analysis of cohort studies
Source: Front Hum Neurosci. 2022 Dec 1;16:899814. doi: 10.3389/fnhum.2022.899814 (PMC9751343; doi:10.3389/fnhum.2022.899814)

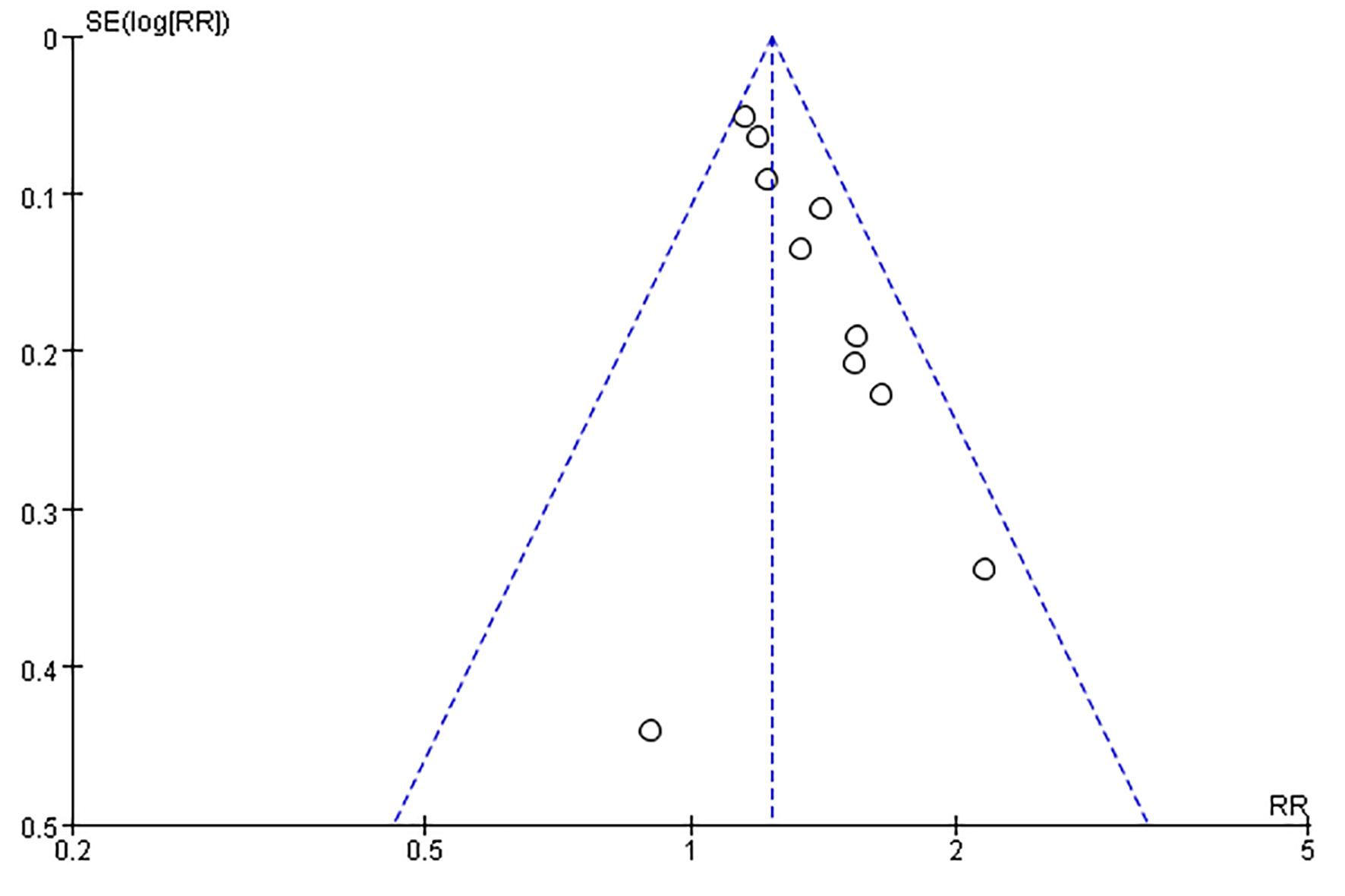

Supplement: Supplementary file 2 [file Image_1.TIF]
