# Supplementary material for: Association between loneliness and dementia risk: A systematic review and meta-analysis of cohort studies
Source: Front Hum Neurosci. 2022 Dec 1;16:899814. doi: 10.3389/fnhum.2022.899814 (PMC9751343; doi:10.3389/fnhum.2022.899814)
Supplement: Supplementary file 3 [file Table_1.DOCX]

Supplementary Tables S1

**Association** **between loneliness and dementia risk: A systematic review and meta-analysis of cohort studies**

Luyao Qiao^a,#^, Zhenyu Tang^a,b,#^, Siqi Zhou^a^, Gege Wang^a^, Jun Min^a,b^, Min Yin^a,b^ and Min Li^a,b,^*

Search strategy

PubMed database search: to February 17, 2022

| 1 | Cohort Studies [Mesh] |
| --- | --- |
| 2 | Longitudinal Studies [Mesh] |
| 3 | Follow-Up Studies [Mesh] |
| 4 | Cohort Studies [T/A] or Longitudinal Studies [T/A] or Follow-Up Studies [T/A] |
| 5 | 1 or 2 or 3 or 4 |
| 6 | Loneliness [Mesh] or Social Isolation [Mesh] or Social Support [Mesh] or Social Participation [Mesh] or Interpersonal Relations [Mesh] or Social Networking |
| 7 | Loneliness [T/A] OR Social Support [T/A] OR Social Isolation [T/A] OR Social Participation [T/A] OR Social Engagement [T/A] OR Social Disengagement [T/A] OR Social Integration [T/A] OR Social Interaction [T/A] OR Social Withdrawal [T/A] OR Social Capital [T/A] OR Social Contact [T/A] OR Social Influence [T/A] OR Social Vulnerability [T/A] |
| 8 | 6 or 7 |
| 9 | Dementia [Mesh] or Alzheimer’s disease [Mesh] or Cognitive Decline [Mesh] |
| 10 | Cognitive Function* OR Cognitive Impairment OR Cognitive Decline OR Cognitive Deficit* [T/A] OR Cognition Loss* [T/A] OR Cognitive Loss* [T/A] OR Cognitive Abilit* [T/A] OR Dement* [T/A] OR Alzheimer* [T/A] OR Cognition [T/A] OR Cognitive Status [T/A] OR Cognitive Change [T/A] OR Cognition Change [T/A] OR Cognitive Performance [T/A] OR Cognitive Disfunction* [T/A] |
| 11 | 9 or 10 |
| 12 | 5 and 8 and 11 |

Search strategy

EMBASE database search: to February 17, 2022

| 1 | ‘cohort studies’/exp OR ‘cohort studies’ OR ‘longitudinal studies’/exp OR ‘longitudinal studies’ OR ‘follow-up studies’/exp OR ‘follow-up studies’ |
| --- | --- |
| 2 | ‘loneliness’/exp OR ‘loneliness’ OR ‘social support’/exp OR ‘social support’ OR ‘social isolation’/exp OR ‘social isolation’ OR ‘social integration’/exp OR ‘social integration’ OR ‘social interaction’/exp OR ‘social interaction’ OR ‘social contact’/exp OR ‘social contact’ |
| 3 | ‘cognitive function*’ OR ‘cognitive impairment’/exp OR ‘cognitive impairment’ OR ‘cognitive decline’/exp OR ‘cognitive decline’ OR ‘cognitive deficit*’ OR ‘dement*’ OR ‘alzheimer*’ OR ‘cognition’/exp OR ‘cognition’ OR ‘cognitive dysfunction*’/exp OR ‘cognitive dysfunction’ |
| 4 | 1 and 2 and 3 |

CNKI database search: to February 17, 2022

| 1 | 队列研究 OR 观察性研究 OR 随访研究 OR 纵向研究 |
| --- | --- |
| 2 | 孤独 OR 孤单 OR 寂寞 OR 社交接触 OR 社交融合 OR 社交支持 OR 社交互动 |
| 3 | 认知障碍 OR 认知衰退 OR 认知缺陷 OR 认知损害 OR 阿尔茨海默病 OR 痴呆 |
| 4 | 1 and 2 and 3 |
