# Supplementary material for: Association between loneliness and dementia risk: A systematic review and meta-analysis of cohort studies
Source: Front Hum Neurosci. 2022 Dec 1;16:899814. doi: 10.3389/fnhum.2022.899814 (PMC9751343; doi:10.3389/fnhum.2022.899814)
Supplement: Supplementary file 4 [file Table_2.DOC]

**Supplementary Tables S2**. Characteristics of studies included in the meta-analysis

| First author year | Location | Sample size, % women | Follow-up period | Mean age (SD), age range | Loneliness measurement | Outcome | No. of cases | Adjustment for covariates | Result (effect size; 95% CI) |
| --- | --- | --- | --- | --- | --- | --- | --- | --- | --- |
| Zhang, et al 1999 | China | 1203, 58% | 10 | NA, 55+ | NA | Incident AD | 124 | Age and sex | 1.63 (0.93, 2.86) |
| Tilvis et al 2004 | Finland | 650, 73% | 10 | NA, 75+ | Do you suffer from loneliness | Incident MCI | 88 | Age, sex, stroke, myocardial infarction, atrial fibrillation, intermittent claudication, hypertension, diabetes mellitus, plasma insulin, C-reactive protein, serum cholesterol, APOE4, serum Ca^++^, and depression | 1.17 (0.76, 1.81) |
| Wilson et al 2007 | United States | 792, 75.7% | 6 | 80.7 (7.1), 50+ | Modified version of the DeJong-Gierveld Loneliness Scale | Incident AD | 76 | Age, sex, level of education, social activity, social network, physical activity, cognitive activity, depressive symptoms, income, race/ethnicity, disability and vascular risk factors | 1.51 (1.07, 2.15) |
| Lobo et al 2008 | Spain | 1654, 55.2% | 3 | 73.6 (8.7), 55+ | Item on loneliness from the GMS | Incident MCI | 208 | Age, sex, educational level, irritability, neurovegetative symptoms, sleep problems, lack of concentration and subjective slowness. | 2.05 (1.31, 3.19) |
| Chen et al 2011 | China | 1307, 43.5% | 7.5 | NA, 65+ | Feeling lonely vs not feeling lonely | Incident dementia | 80 | Age and sex | 0.90 (0.38, 2.13) |
| Holwerda et al 2014 | The Netherlands | 2173, 63.1% | 3 | NA, 65-86 | Do you feel lonely or do you feel very lonely? | Incident dementia | 158 | Age, sex, level of education, marital status, living situation (alone), depression, cardiovascular and other medical conditions, cognitive function, ADL/IADL and social support. | 1.64 (1.05, 2.56) |
| Rawtaer et al 2017 | Singapore | 1601, 64.5% | 8 | 64.9 (6.8), 55+ | Do you feel that at the present moment you are not at all lonely or fairly lonely or very lonely? | Incident MCI-dementia | 163 | Age, sex, ethnicity, smoking, alcohol consumption, dyslipidemia, hypertension, diabetes, obesity, stroke/heart disease, APOE-e4 allele carrier, depression. physical activities, social activities, productives, living status, marital status and satisfaction with life. | 1.02 (0.66, 1.57) |
| Zhou et al 2018 | China | 7867, 54.9% | 3 | 83.1 (10.9), 65+ | Do you often feel lonely? | Incident dementia | 393 | Age, sex, type of area (rural), level of education, living situation, marital status, social support, institutionalization, exercise, smoking, alcohol consumption, nut and milk intake, stroke, cerebrovascular diseases, hypertension, diabetes, cognitive function and ADL. | 1.15 (1.04, 1.28) |
| Shibata et al 2020 | Japan | 1141, 55.3 | 5 | 73.4 (6.2) | The six-item De Jong Gierveld Loneliness Scale | Incident dementia | 114 | Age, sex, educational level, employment status, current smoking, current drinking, regular exercise, daily life activity, BMI, hypertension, diabetes, electrocardiogram abnormalities, past history of cardiovascular disease, cancer, other chronic diseases, and depression | 1.22 (1.02, 1.46) |
| Rafnsson et al 2020 | England | 1. 55.7% | 6.25 | 66.0 (9.4) | The three-item, short form of the Revised UCLA loneliness scale | Incident dementia | 220 | Age, sex, education, wealth, hypertension, diabetes, stroke, coronary heart disease, cancer, mobility,depression, marital status,and social isolation | 1.33 (1.02, 1.73) |
| Luchetti et al 2020 | Europe | 14411, 54.7% | 11 | 63.6 (9.3) | How often have you experienced the following feelings over the last week: I felt lonely? | Incident MCI | 525 | Age, sex, education level, BMI, hypertension, diabetes, physical activity, smoking, and depressive symptoms | 1.15 (1.01, 1.32) |
| Sundstrom et al 2020 | Sweden | 1905, 52.6% | 11 | 71.5 (9.3) | Do you often feel lonely? | Incident dementia, AD, and VaD | 856 | Age, sex,education, marital status, smoking, alcohol use, previous cardiovascular disorders, and depressive symptoms | 1.53 (1.02, 2.27) |
| Sutin et al 2020 | United States | 12030, 60% | 10 | 67.3 (10.45) | The three-item, short form of the Revised UCLA loneliness scale | Incident dementia | 1104 | Age, sex, race, education, hypertension, diabetes, smoking, physical activity, BMI, social isolation, and depressive symptoms | 1.19 (1.05, 1.33） |
| Salinas et al 2022 | United States | 2308, 56% | 10 | 73 (9) | CES-D | Incident dementia | 329 | Age, sex, education, depressive symptoms, social isolation and additionally adjusts for antidepressant medication use | 1.54 (1.06, 2.24) |
| Freak-Poli (RS) et al 2022 | The Netherlands | 4514, 57% | 14 | 71.3 (7.4) | CES-D | Incident dementia | 521 | Age, sex, education, smoking status, alcohol, hypertension, hypercholesterolemia, diabetes, activities of daily living disability index, BMI and major depressive disorder | 1.40 (1.13, 1.72) |
| Freak-Poli (SNAC-K) et al 2022 | Sweden | 2112, 36% | 10 | 71.6 (9.9) | CES-D | Incident dementia | 301 | Age, sex, education, smoking status, alcohol, hypertension, hypercholesterolemia, diabetes, activities of daily living disability index, BMI and major depressive disorder | 2.15 (1.11, 4.16) |

Abbreviations: NA, not available; AD, Alzheimer’s disease; MCI, mild cognitive impairment; VaD, vascular dementia; ADL, activities of daily living; IADL, instrumental activities of daily living; BMI, body mass index; CES-D, The Center for Epidemiologic Studies Depression Scale; SNAC-K, Swedish National study on Aging and Care in Kungsholmen; RS, Rotterdam Study.
