# Supplementary material for: Association between loneliness and dementia risk: A systematic review and meta-analysis of cohort studies
Source: Front Hum Neurosci. 2022 Dec 1;16:899814. doi: 10.3389/fnhum.2022.899814 (PMC9751343; doi:10.3389/fnhum.2022.899814)
Supplement: Supplementary file 5 [file Table_3.DOC]

Supplementary Tables S3 Quality assessment of included studies by Newcastle-Ottawa Scale.

| Reference, publication (year) | Selection | | | |  | Comparability | |  | Outcome | | | Score |
| --- | --- | --- | --- | --- | --- | --- | --- | --- | --- | --- | --- | --- |
|  | Representativeness of exposed cohort | Selection of non-exposed cohort | Exposure Ascertainment | Outcome present at start of study |  | Study controls for age | Study controls for any additional important factor |  | Assessment of Outcome | Length of follow-up | Adequacy of follow-up |  |
| Zhang et al (1999) | ★ | ★ |  | ★ |  | ★ |  |  | ★ | ★ | ★ | 7 |
| Tilvis et al (2004) | ★ | ★ |  | ★ |  | ★ | ★ |  | ★ | ★ |  | 7 |
| Wilson et al (2007) |  | ★ | ★ | ★ |  | ★ | ★ |  | ★ | ★ | ★ | 8 |
| Lobo et al (2008) | ★ | ★ |  | ★ |  | ★ | ★ |  | ★ |  |  | 6 |
| Chen et al (2011) | ★ | ★ |  | ★ |  | ★ | ★ |  | ★ | ★ |  | 7 |
| Holwerda et al (2014) | ★ | ★ |  | ★ |  | ★ | ★ |  | ★ |  |  | 6 |
| Rawtaer et al (2017) | ★ | ★ |  | ★ |  | ★ | ★ |  | ★ | ★ | ★ | 8 |
| Zhou et al (2018) | ★ | ★ |  | ★ |  | ★ | ★ |  | ★ |  |  | 6 |
| Shibata et al (2020) | ★ | ★ | ★ | ★ |  | ★ | ★ |  | ★ | ★ |  | 8 |
| Rafnsson et al (2020) | ★ | ★ | ★ | ★ |  | ★ | ★ |  |  | ★ |  | 7 |
| Luchetti et al (2020) | ★ | ★ |  | ★ |  | ★ | ★ |  | ★ | ★ | ★ | 8 |
| Sundström et al (2020) | ★ | ★ | ★ | ★ |  | ★ | ★ |  | ★ | ★ |  | 8 |
| Sutin et al (2020) |  | ★ | ★ | ★ |  | ★ | ★ |  | ★ | ★ |  | 7 |
| Salinas et al (2022) |  | ★ | ★ | ★ |  | ★ | ★ |  | ★ | ★ | ★ | 8 |
| Freak-Poli (RS) et al (2022) | ★ | ★ |  | ★ |  | ★ | ★ |  | ★ | ★ | ★ | 8 |
| Freak-Poli (SNAC-K) et al (2022) | ★ | ★ |  | ★ |  | ★ | ★ |  | ★ | ★ | ★ | 8 |
